# Identification and Characterization of the Masculinizing Function of the *Helicoverpa armigera Masc* Gene

**DOI:** 10.3390/ijms22168650

**Published:** 2021-08-11

**Authors:** Zhongyuan Deng, Yakun Zhang, Yalu Li, Kaiyuan Huang, Xuewei Chen, Min Zhang, Jinyong Huang, Xinzhi Ni, Xianchun Li

**Affiliations:** 1 School of Agricultural Sciences, Zhengzhou University, Zhengzhou 450001, China; dengzhongyuan@outlook.com (Z.D.); pirna@foxmail.com (Y.L.); aauaaa@foxmail.com (K.H.); chen_xw@zzu.edu.cn (X.C.); zhangmin753@gmail.com (M.Z.); jinyhuang@zzu.edu.cn (J.H.); 2 State Key Laboratory for Biology of Plant Diseases and Insect Pests, Institute of Plant Protection, Chinese Academy of Agricultural Sciences, Beijing 100193, China; zhangyakun7@bjfu.edu.cn; 3 Crop Genetics and Breeding Research Unit, USDA-ARS (United States Department of Agriculture-Agricultural Research Service), Tifton, GA 31793, USA; xinzhi.ni@usda.gov; 4 Department of Entomology, University of Arizona, Tucson, AZ 85721, USA

**Keywords:** chromosome, development, *doublesex*, sex determination, sperm bundle, testis fusion

## Abstract

The *Masculinizer* (*Masc*) gene has been known to control sex development and dosage compensation in lepidopterans. However, it remains unclear whether its ortholog exists and plays the same roles in distantly related lepidopterans such as *Helicoverpa armigera*. To address this question, we cloned *Masc* from *H. armigera* (*HaMasc*), which contains all essential functional domains of BmMasc, albeit with less than 30% amino acid sequence identity with BmMasc. Genomic PCR and qPCR analyses showed that *HaMasc* is a Z chromosome-linked gene since its genomic content in males (ZZ) was two times greater than that in females (ZW). RT-PCR and RT-qPCR analyses revealed that *HaMasc* expression was sex- and stage-biased, with significantly more transcripts in males and eggs than in females and other stages. Transfection of a mixture of three siRNAs of *HaMasc* into a male embryonic cell line of *H. armigera* led to the appearance of female-specific mRNA splicing isoforms of *H. armigera* *doublesex* (*Hadsx*), a downstream target gene of *HaMasc* in the *H. armigera* sex determination pathway. The knockdown of *HaMasc,* starting from the third instar larvae resulted in a shift of *Hadsx* splicing from male to female isoforms, smaller male pupa and testes, fewer but larger/longer spermatocytes and sperm bundles, delayed pupation and internal fusion of the testes and follicles. These data demonstrate that *HaMasc* functions as a masculinizing gene in the *H. armigera* sex-determination cascade.

## 1. Introduction

The sex of an individual insect is determined genetically upon fertilization and developmentally upon embryogenesis [1,2,3]. The developmental determination of sex is regulated by a cascade of genes that act upon one another to form the sex-determination pathway in a given insect species [1,2,3,4]. The pathway is triggered or initiated by a primary signal derived from the chromosomal makeup of a fertilized egg, such as the X chromosome/autosome ratio in *Drosophila melanogaster* [5] and the W chromosome-linked *Feminizer* piRNA in the *Bombyx mori* [6]. The primary signal is transduced, often via alternatively splicing, to two terminal double-switch genes known as *doublesex* (*dsx*) and *fruitless* (*fru*) that specify the sexual fate of a fertilized egg [1,4,7,8]. Between the primary signal and the two terminal switch genes stands a key gene that undertakes a splicing-mediated autoregulatory feedback loop [7]. In the presence or absence of the primary signal, a few alternatively spliced regulatory genes undergo regulated splicing in one sex, and default splicing in the other sex, leading to sex differentiation [4,7].

Work on the sex determination pathway among different insect species from Diptera, Coleoptera, Hymenoptera, and Lepidoptera support Wilkins’s “bottom-up” theory: between-taxa or species-divergence sequentially elevates from the terminal end to the beginning point of the pathway [7,9]. For example, *dsx* and *fru* are the common terminal switch genes in all insects, whereas the upstream key gene is *Sex-lethal* (*Sxl*) in *Drosophila melanogaster*, *transformer* (*tra*) in many other dipterans, coleopterans, and hymenopterans, *Feminizer* (*fem*) in *Apis mellifera*, and the IGF-II mRNA-binding protein (*BmImp*) in *Bombyx mori* [7]. The most diverse step is the initiating primary signal, which can be a female-determining factor such as a double dose of the X-linked signal elements in *D. melanogaster* [5], heterozygosity of the *complementary sex determiner* (*csd*) gene in honeybees [10], or a W chromosome-linked Piwi-interacting RNA (piRNA), named *Feminizer* piRNA (*Fem* piRNA), in *B. mori* [6]. In contrast, the primary signal can also be a dominant male-determining factor (M factor) located on a Y chromosome or a homomorphic sex-determining chromosome in many other species, including mosquitoes, non-*Drosophila* flies, beetles, and true bugs [11,12,13,14,15]. Among the identified M factors are *Nix* in *Aedes aegypti* [16], *gYG2* (*An. gambiae* Y Gene 2, renamed *Yob*) in *Anopheles gambiae* [17,18], *Guy1* in *Anopheles stephensi* [19], *Mdmd* in *Musca domestica* [20], and *MoY* in *Ceratitis capitata* [21]. These M factors are not related to each other, indicating rapid evolution of the primary signals [8].

Relatively much less is known about the sex-determination pathway in the ZW order Lepidoptera. In contrast to XY insects, in Lepidoptera, males are homogametic (ZZ), and females are heterogametic (ZW) [22,23]. Almost all information about sex-determination cascade in this order comes from studies in the model lepidopteran *B. mori.* An aspect of the uniqueness of the *B. mori* sex-determination cascade is that it has both Fem piRNA, a female determinant from the W chromosome, and *Masculinizer* (*Masc*), a male determinant from the Z chromosome [6,24]. The absence of the W chromosome, and thus, of the primary signal Fem piRNA in males permits *B. mori Masc* (*BmMasc*) to induce male-specific splicing of *BmImp*, the autoregulatory key gene [25]. The resultant male isoform of *BmImp* interacts with the *B. mori* P-element somatic inhibitor (BmPSI), and possibly, BmSPX as well, to stimulate male-specific splicing of *Bmdsx* [4,26,27,28]. In contrast, the presence of the primary signal Fem piRNA in females leads to the default female-specific splicing of *Bmdsx* because a complex of Fem piRNA with BmPIWI (= BmSIWI) and BmAsh2 [29] target *BmMasc* mRNA for degradation to a much lower level, which is insufficient to induce the regulated male-specific splicing of *BmImp* and then *Bmdsx* [6].

The question to be addressed here is whether a homolog of *BmMasc* exists and plays the same role in the distantly related *Helicoverpa armigera*, a noctuid lepidopteran of economic importance. We initiated this study by a BLAST search of an *H. armigera* larvae transcriptome, followed by the PCR cloning of the full-length cDNA sequence of *HaMasc* (the *H. armigera* homolog of *BmMasc*), quantitative PCR (qPCR) analysis of *HaMasc* copies, RT-qPCR (quantitative reverse transcriptase-PCR) analysis of *HaMasc* expression, and the RNAi (RNA interference) knockdown of *HaMasc.* The findings from this study demonstrate that *HaMasc*, like *BmMasc*, is a Z chromosome-linked male determinant required for the regulated male-specific splicing of *dsx* and the normal development of testis.

## 2. Results

### 2.1. Cloning and Identification of HaMasc

**Figure 1:**
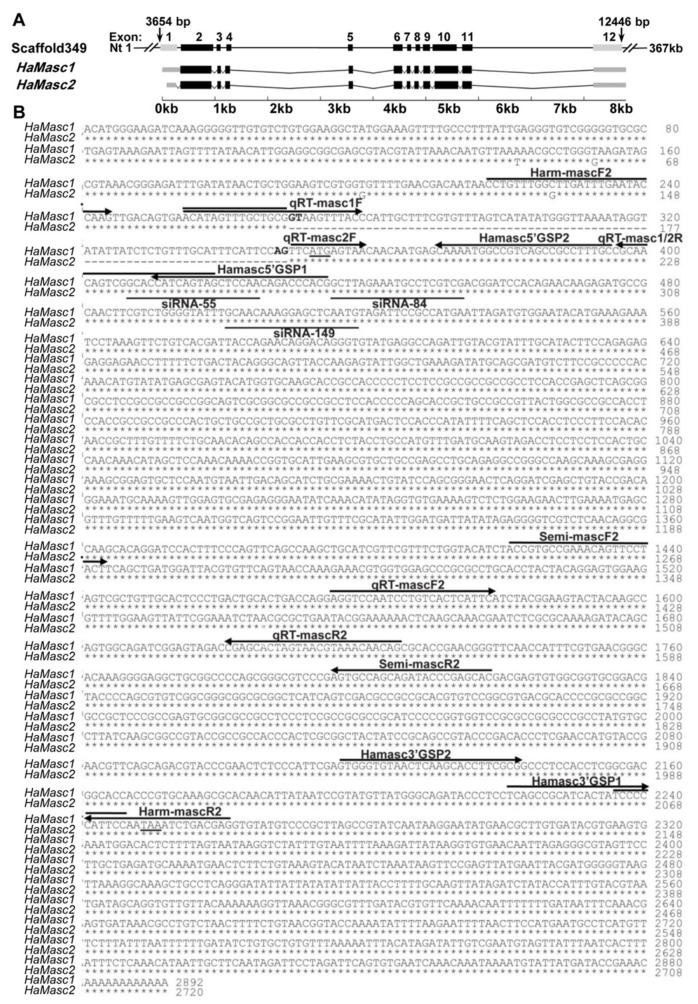
Identification and characterization of *HaMasc.* (**A**) Genomic structure of *HaMasc* gene. Black boxes represent the ten exons for the common ORF of the two *HaMasc* transcripts. Gray boxes depict the exons for the 5′ and 3′ UTR of the *HaMasc* transcripts. The spaces between boxes indicate introns. The size of the putative exons and introns is drawn to scale. (**B**) cDNA nucleotide sequence of *HaMasc* transcripts. The two transcripts of *HaMasc*, called *HaMasc1* and *HaMasc2*, are aligned with asterisks (*) and dashes (-) depicting the conserved nucleotides and indels (insertions/deletions) between the two transcripts, respectively. The start (ATG) and stop (TGA) codons are underlined. The target regions of the three siRNA are indicated by lines and the corresponding siRNA names. The annealing directions and positions of the primers used for the 5′ RACE, 3′ RACE, and full-length cDNA cloning of *HaMasc*, as well as for RT-PCR and RT-qPCR analyses of *HaMasc*, are depicted with arrowed lines and the corresponding primer names. When two primers or siRNAs partially anneal to or target the same positions, they have an overlapping line.

Two full-length cDNA sequences of *H. armigera* homolog of *BmMasc* (NP_001296506.1), named *HaMasc1* (GenBank accession number: MH844486) and *HaMasc2* (GenBank accession number: MH844487) (Figure 1A), were obtained by TBLASTN retrieval of the contig asmbl_65189 from an *H. armigera* transcriptome dataset [30], followed by the RT-PCR cloning of contig asmbl_65189 and the amplification of the 5′ and 3′ ends of *HaMasc* through 5′ and 3′ RACE. The two cDNA sequences shared a common open reading frame (ORF) of 1896 bp encoding 632 amino acid residues and a common 3′-untranslated region (UTR) of 641 bp but differed in the size of the 5′ UTR (Figure 1A). The mapping and alignment of the two cDNA sequences to their genomic sequence in scaffold 349 of the *H. armigera* genome show that the *HaMasc* gene contains 12 exons and 11 introns (Figure 1B). Exons 1 and 12 belong to *HaMasc’s* 5′ UTR and 3′ UTR, respectively, whereas Exons 2–11 compose *HaMasc’s* ORF (Figure 1B). The 5′ UTR difference between *HaMasc1* and *HaMasc2* resulted from the retention of Intron 1 in *HaMasc1* and the absence of the 5′–92 bp sequence of exon 1 in *HaMasc2* (Figure 1).

**Figure 2:**
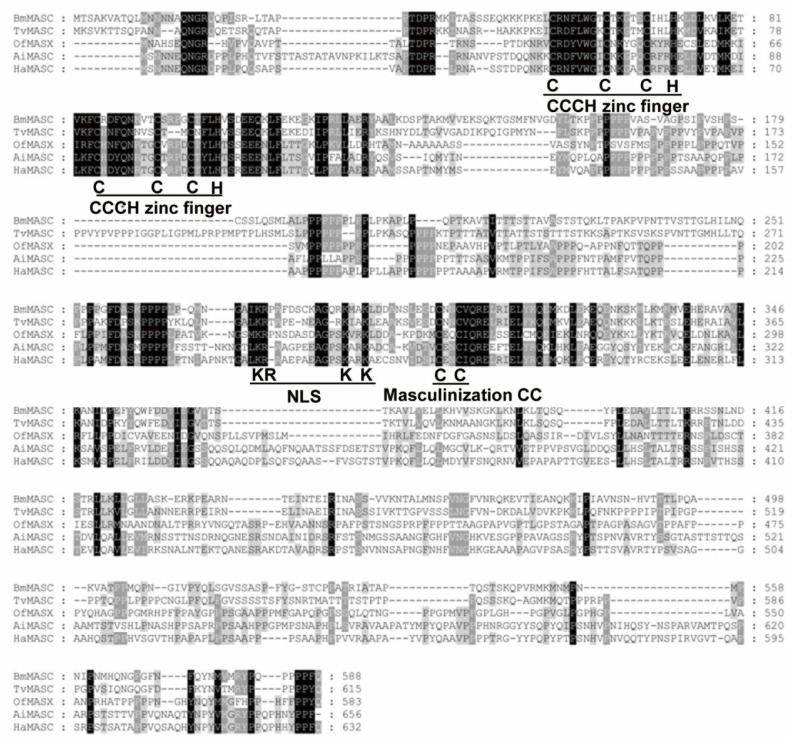
Amino acid alignment of HaMasc with four lepidopteran Masc proteins. Amino acids missed in any of the five Masc proteins are depicted by dashes. Amino acids conserved among all five Masc proteins are shaded in black, whereas those conserved among two to four Masc proteins are shaded in gray. The conserved tandem CCCH-type zinc fingers, nuclear localization signal (NLS), and masculinization CC are underlined. BmMasc = *Bombyx mori* Masc; TvMasc = *Trilocha varians* Masc; OfMasc = *Ostrinia fumacalis* Masc; AiMasc = *Agrotis ipsilon* Masc; HaMasc = *Helicoverpa armigera* Masc.

**Figure 3:**
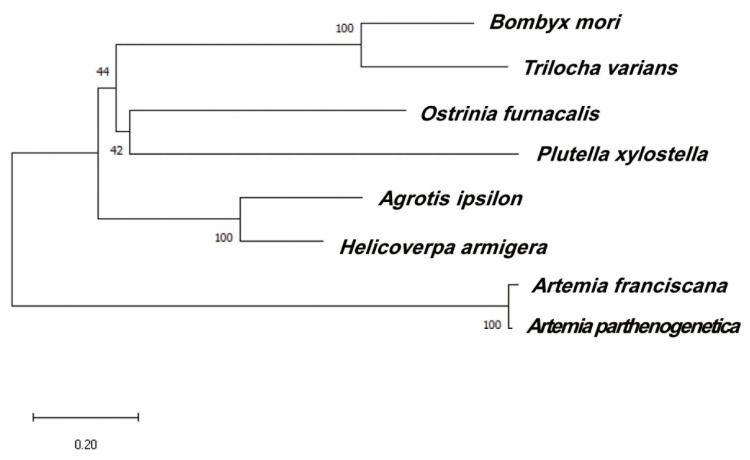
Phylogenetic relationship among known lepidopteran Masc proteins. The neighbor-joining tree of lepidopteran Masc proteins with two brine shrimp (*Artemia* species) Masc proteins as the outgroup was drawn with branch length proportional to the inferred amino acid changes. The scale rule for amino acid changes is shown at the bottom. The bootstrap values are shown at the base of each internal branch.

While the deduced HaMasc protein shares only a 21.14% to 62.57% identity with Masc homologs from *T. variants* [31], *B. mori* [6], *O. furnacalis* [32], *A. ipsilon* [33], and *Plutella xylostella* [34] (Table 1), HaMasc, like the above Masc proteins, contains two tandem CCCH-type zinc finger domains, one nuclear localization signal (NLS) and one CC masculinization motif (Figure 2), which are essential for the functions of BmMasc [6]. Phylogenetic analysis grouped *HaMasc* and *AiMasc* in the same terminal lineage, consistent with the fact that *H. armigera* and *A. ipsilon* belong to the same family of Noctuidae (Figure 3).

### 2.2. Chromosomal Assignment of HaMasc to Z Chromosome

**Figure 4:**
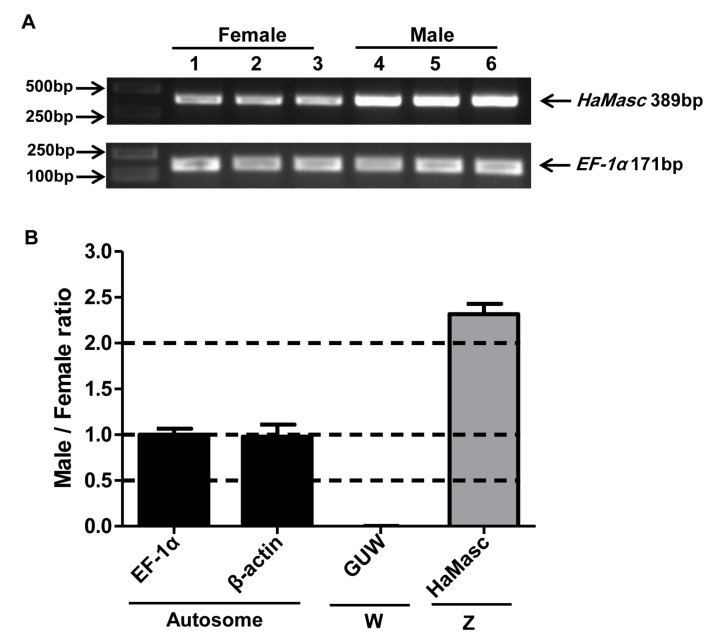
Chromosomal affiliation of *HaMasc.* (**A**) Genomic PCR gel analysis of *HaMasc* and *EF-1α*. Genomic DNAs extracted from three female pupa (numbered 1, 2, 3) and three male pupa (4, 5, 6) were used as the template to PCR-amplify *HaMasc* and *EF-1α*, respectively. A representative gel image of each gene is presented in (**A**). (**B**) Genomic qPCR analysis of *HaMasc*, *W-linked GUW1*, and autosomal genes *EF-1α* and *β-actin.* The templates (three male DNA samples and three female DNA samples) used for qPCR are the same as for PCR gel analysis.

Genomic PCR gel analysis showed that the band intensity of *HaMasc* amplified from male (AA + ZZ = 2 copies of each autosome + 2 copies of Z chromosome) pupa was roughly two times of that from female (AA + ZW = 2 copies of each autosome + 1 copy of Z chromosome/1 copy of W chromosome) pupa, whereas the band intensity of the autosomal gene *EF-1α* was equal between the male and female pupa (Figure 4A). Further genomic qPCR analysis revealed that the male copy/female copy ratio was 0.0023 for the W chromosome gene *GUW1* [35], 1.00 for the autosomal gene *EF-1α*, 0.98 for the autosomal gene *β-actin*, and 2.32 for *HaMasc* (Figure 4B). These data confirm that the *HaMasc* gene is located on Z chromosome.

### 2.3. Sex-, Stage- and Tissue-Specific Expression of HaMasc

**Figure 5:**
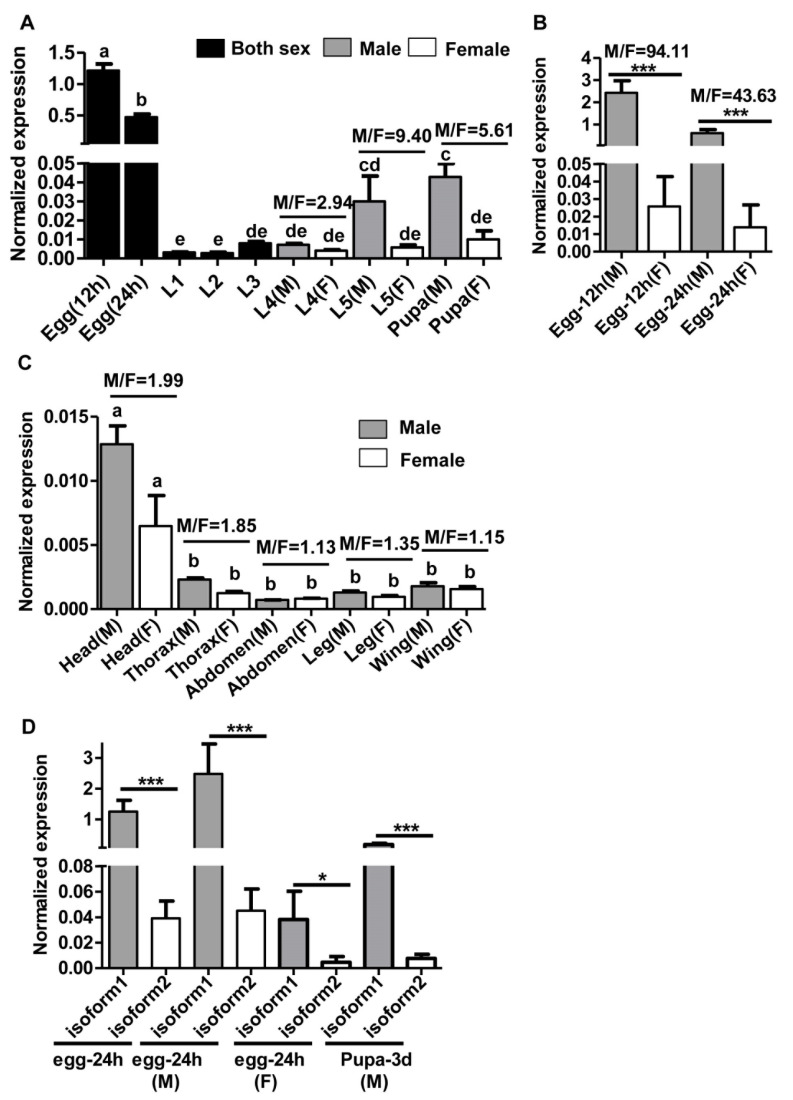
Expression profiles of *HaMasc.* (**A**) RT-qPCR analyses of *HaMasc* in unsexed (eggs to the third instar larvae (L3)) and sexed (L4 (the fourth instar larvae) to pupa) development stages. (**B**) RT-qPCR analyses of *HaMasc* in sexed eggs (12 h and 24 h). (**C**) RT-qPCR analysis of *HaMasc* in different body parts of newly emerged male (M) and female (F) adults. (**D**) RT-qPCR analysis of the two *HaMasc* isoforms (*HaMasc1* and *HaMasc2*) in eggs (24 h, unsexed) and male pupa. Bars in (**A**–**D**) are means ± SE of normalized expression of *HaMasc* based on at least three biological replicates. Bars with different letters such as a, b, c, d, and e in Figure 5A,C (*p* < 0.05, one-way ANOVA followed by Tukey’s HSD test) are significantly different. Bar pairs with one asterisk (*p* < 0.05, independent *t*-test) and three asterisks (*p* < 0.001, independent *t*-test) in Figure 5B,D are significantly and extremely different, respectively.

We analyzed the expression of *HaMasc* at different developmental stages of both sexes (undistinguishable before the fourth instar larvae (L4)) (Figure 5A,B) and in different body parts of newly-emerged male and female adults (Figure 5C) by RT-qPCR analyses of the common region of the two *HaMasc* isoforms using the primer pair qRT-mascF2 and qRT-mascR2 for RT-qPCR (Table S1; Figure 1B). *HaMasc* displayed the highest expression in 12 h eggs, followed by 24 h eggs, male pupa, male L5, L3-L4, female L5, female pupa, and L1-L2 (Figure 5A). At stages when test insects were sexed by the presence of the W chromosome-specific *GUW* marker (12 and 24 h eggs) (Deng et al.2020), the presence of testes (L4-L5) or the relative distance between the reproduction and excretion holes (pupa) [36], *HaMasc* expression was 94.11- (12 h eggs), 43.63- (24 h eggs), 2.94- (L4), 9.4- (L5), and 5.61-fold (pupa) greater in males than that in females (Figure 5A,B), respectively. The difference in *HaMasc* expression between male and female eggs and pupa was significant but the difference was not significant among L1, L2, L3, L4, female L5, and female pupa (Figure 5A,B).

Newly emerged male adults had a roughly similar level of *HaMasc* expression with L3, L4, female L5, and female pupa, whereas female adults exhibited a lower level of *HaMasc* expression than any other developmental stages (compare Figure 5C vs. Figure 5A). Adult head tissue had a significantly higher level of *HaMasc* expression than the other four adult tissues examined, including thorax, abdomen, leg, and wing tissues (Figure 5C). In all five body parts, *HaMasc* expression was numerically (M/F ratio ranged from 1.13 to 1.99) higher in males than in females.

To address which of the two *HaMasc* isoforms plays a more important role in the *H. armigera* sex-determination pathway, we designed two pairs of isoform-specific primers (see qRT-masc1F/qRT-masc1/2R and qRT-masc2F/qRT-masc1/2R in Table S1 and Figure 1B) to RT-qPCR-analyze the expressions of the two isoforms of *HaMasc* transcripts in 24-h-old eggs (sexed and unsexed) and 3-d-old male pupae, two stages with the highest expression of *HaMasc* (Figure 5A,B). As shown in Figure 5D, the expression levels of *HaMasc* isoform 1 (*HaMasc1*) in 24-h-old unsexed eggs, 24-h-old male eggs, 24-h-old female eggs, and 3-d-old male pupae were 32.17-, 55.17-, 8.32-, and 23.81-fold greater than those of *HaMasc* isoform 2 (*HaMasc2*), respectively, suggesting that *HaMasc1* is the dominant isoform and plays a more important role than *HaMasc2.*

### 2.4. Suppressing HaMasc Caused Female-Specific Splicing of Hadsx

**Figure 6:**
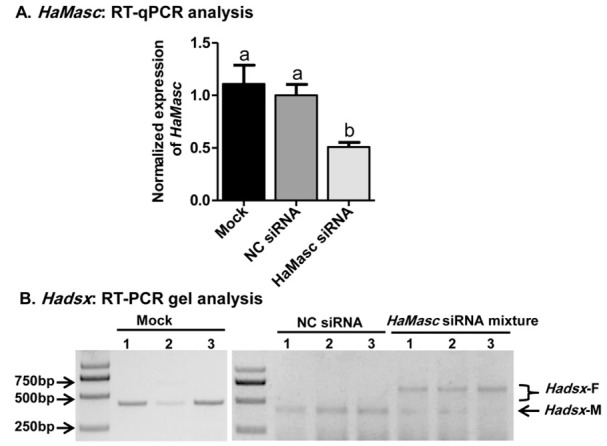
Effects of *HaMasc* siRNA mixture on the expression of *HaMasc* and *Hadsx* in the *H. armigera* male embryo cell line QB-Ha-E-1. (**A**) RT-qPCR analysis of *HaMasc* expression, and (**B**) RT-PCR analysis of sex-specific splicing of *Hadsx*. Mock was the female and male individual without treatment. Bars in (**A**) are means ± SE of normalized expression of *HaMasc* based on at least three biological replicates of three independent transfections of each siRNA. Bars with different letters (a, b in Figure 6A) are significantly different at *p* < 0.05 (one-way ANOVA followed by Tukey’s HSD test).

Whether a candidate gene functions as an upstream effector gene in the sex-determination pathway of an insect species is dependent on whether knockout/knockdown or overexpression shifts the sex-specific splicing of the terminal double-switch gene *dsx* from the male-specific to female-specific isoforms or vice versa. The knockout-/knockdown-triggered shift from the male-specific to female-specific *dsx* splicing isoforms has been used as the essential evidence to verify the masculinizing function of the lepidopteran *Masc* genes examined so far [31,32,33,34]. To observe the effects of *HaMasc* on *Hadsx* splicing, we used RNA interference (RNAi) to knock down *HaMasc* in the male embryo cell line QB-Ha-E-1 developed from 24-h-old eggs. QB-Ha-E-1 cells were transfected with a mixture of 3 *HaMasc* siRNAs (Figure 1) or a negative control siRNA (NC siRNA), each at 20 pmol of siRNA per mL of medium. Relative to NC siRNA, *HaMasc* siRNAs significantly reduced *HaMasc* transcript abundance (52.82% reduction) (Figure 6A). In addition, *HaMasc* siRNAs induced a *Hadsx* splicing shift from male-specific isoforms to female-specific isoforms plus a reduced abundance of male-specific isoforms (Figure 6B).

### 2.5. Suppressing HaMasc Retarded Male Growth and Testis Development

**Figure 7:**
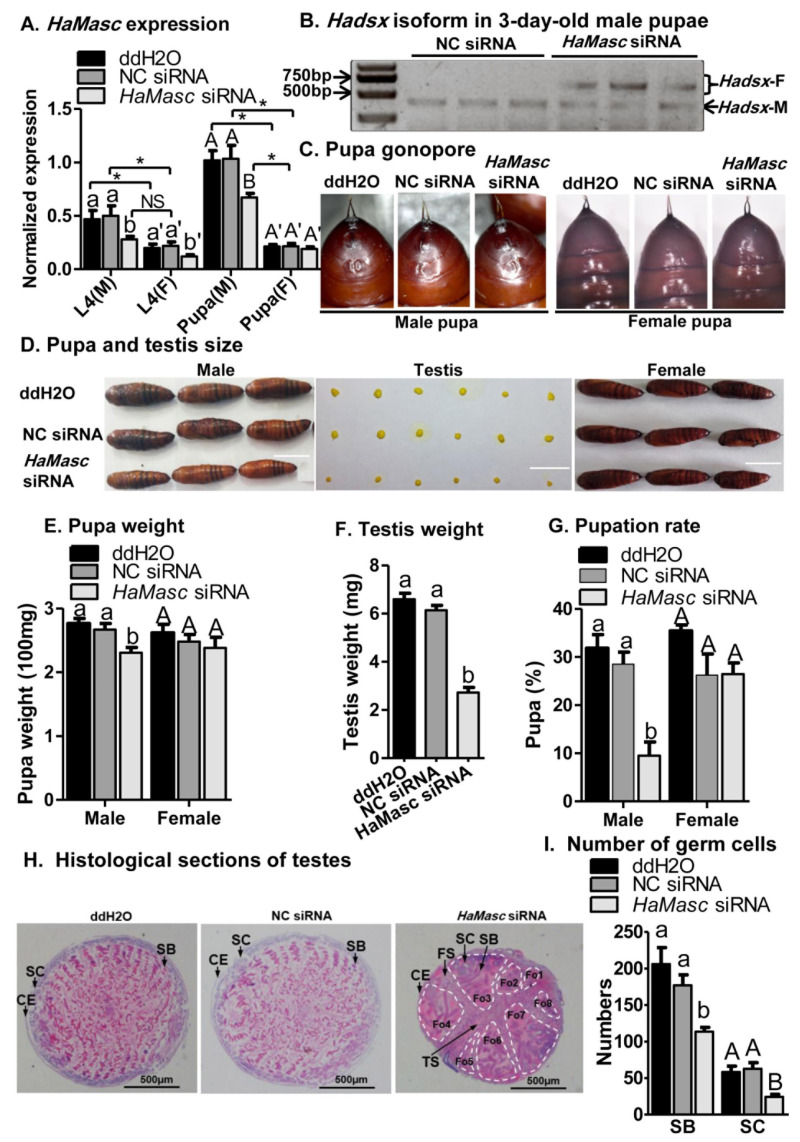
Impacts of *HaMasc* siRNA mixture on *H. armigera* larvae. *HaMasc* expression (**A**; M = male, F = female), splicing of *Hadsx* in 3-day-old male pupae (**B**), pupa gonopore (**C**), pupa and testis size (**D**), pupa weight (**E**), testis weight (**F**), pupation rate 2 weeks after delivery of siRNAs (**G**), testis development and structure and, (**H**) germ cell number (**I**). Bars in (**A**,**E**–**G**,**I**) are means ± SE of normalized expression of *HaMasc*, pupa weight, testis weight, pupation rate, and germ cell number, respectively. Bars with different letters (a, b, A, B, A’ in Figure 7A,E–G,I) are significantly different at *p* < 0.05 (one-way ANOVA followed by Tukey’s HSD test). Photographs of representatives of 3-day-old pupa (**D**), their gonopore (**C**), and male testes (**D**) from each treatment group (ddH_2_O, NC siRNA, *HaMasc* siRNA mixture) were taken with a camera (**D**: bar = 1 cm). Hematoxylin and eosin (HE) staining of histological sections of the testis from 3-day-old male pupa (**H**: bar = 500 μm) shows that the internal fusion process of two bilaterally symmetrical testes into one single testis was completed in ddH_2_O and NC siRNA pupa but not in *HaMasc* siRNAs pupa, as manifested by the presence of septa between the two testes (TS in **H**) and between the four follicles within each testis (FS in **H**) in *HaMasc* siRNAs pupa but not in ddH_2_O and NC siRNA pupa. The notable eight follicles (Fo 1–8) in *HaMasc* siRNAs pupa are surrounded by white dotted lines. FS = follicle septa, TS = testis septa, CE = coating epithelium, SC = spermatocytes, SB = sperm bundle. Bar pairs with one asterisk (*p* < 0.05, independent *t*-test).

To examine the effects of *HaMasc* on testis development and larval growth, we suppressed *HaMasc* in *H. armigera* larvae by a combination of injection and feeding of ddH_2_O, NC siRNA, or *HaMasc* siRNAs. Relative to ddH_2_O and NC siRNA, *HaMasc* siRNAs not only significantly decreased *HaMasc* expression in male larvae (44.22% reduction), female larvae (45.78% reduction), and 3-d-old male pupae (32.84% reduction) (Figure 7A), but also induced the appearance of the female-specific splicing isoforms of *Hadsx* in 3-d-old male pupae (Figure 7B). A *t*-test showed that male larvae treated with ddH_2_O or NC siRNA had a significantly higher expression of *HaMasc* than the corresponding female larvae (Figure 7A). However, *HaMasc* siRNA reduced the expression of *HaMasc* in male larvae to a similar level of *HaMasc* in female larvae treated with dd H_2_O or NC siRNA (Figure 7A). While *HaMasc* siRNAs did not affect the gonopore of male pupae (Figure 7C), it significantly reduced the body and testis sizes of male pupae (Figure 7D) as well as the weights of male pupae (13.65% reduction, Figure 7E) and testis (55.61% reduction, Figure 7F). Treatments with *HaMasc* siRNAs also significantly extended the duration from the third instar larvae to pupa as the percentage of larvae reaching pupal stage on Day 14 post-injection was 10% for larvae treated with *HaMasc* siRNAs, but 29% for larvae with NC siRNA, and 32% for larvae with ddH_2_O in male individuals (Figure 7G). In contrast, no differences in *HaMasc* expression in 3-d-old female pupae (Figure 7A), female pupa gonopore (Figure 7B), female pupae body size (Figure 7D), female pupae body weight (Figure 7E), and female pupation rate on Day 14 (Figure 7G) were observed among the three treatment groups. The differences in the pupation rate on Day 14 were not a simple reflection of reduced survival by *HaMasc* siRNAs (Figure S1).

Externally, 3 d after pupation, in all three injection/feeding groups, the process of fusing two bilaterally symmetrical testes into one single testis was completed (Figure 7D). The histological section, however, showed that such was true only for ddH_2_O-and-NC-siRNA-treated pupa, but not for *HaMasc*-siRNAs-treated pupa (Figure 7H). This was evidenced by the presence of septa between the two testes (TS in Figure 7H) and between the four follicles within each testis (FS in Figure 7H) in the *HaMasc*-siRNA-treated pupa, but not in the ddH_2_O-and-NC-siRNA-treated pupa. Obvious twisting was observed in ddH_2_O-and-NC siRNA-treated pupa, but not in *HaMasc*-siRNAs-treated pupa. Spermatocytes (SC) were larger and sperm bundles (SB) were longer in *HaMasc*-siRNAs-treated pupa than in ddH_2_O-and-NC-siRNA-treated pupa. There were significantly fewer SC and SB in *HaMasc*-siRNA-pupa than in ddH_2_O-and-NC-siRNA-pupa (Figure 7H,I).

## 3. Discussion

*BmMasc*, the target gene of the primary signal Fem piRNA in the *B. mori* sex determination cascade [6,37], is expected to evolve rapidly according to Wilkins’s “bottom-up” theory [7,9], and thus, may or may not have a recognizable homolog in *H. armigera* since the two lepidopterans belong to two distant families [38,39]. The data obtained in this study confirm that the *H. armigera* homolog of *BmMasc*, *HaMasc*, does exist in the *H. armigera* genome and transcriptome (Figure 1), although its amino acid identity (only 25%) with BmMasc (Table 1; Figure 2) fails to meet the widely accepted 30% criterion for homology identification [40]. One reason for this homology assignment is that HaMasc contains all known functional domains of BmMasc including two tandem CCCH-type zinc fingers, one bipartite nuclear localization signal, and one masculinization CC (Figure 2), which are required for masculinizing and/or dosage compensation activity of BmMasc [41,42,43]. Another is that Mascs from *Agrotis ipsilon* (AiMasc) [33], *Ostrinia fumacalis* (OfMasc) [32], and *Plutella xylostella* (PxMasc) [34] play a similar male-determining role in the three species, even though their amino acid sequence identities with BmMasc are even lower than that of HaMasc with BmMasc, ranging from 19.74% to 22.48% (Table 1).

Other than low amino acid sequence identity, as described above, the rapid evolution of *Masc* genes is also evidenced by their variations in gene structure and alternative splicing among and within families. The exon number varies from 10 in *B. mori Masc* [44] to 11 in *A. ipsilon Masc* [33], 12 in *H. armigera Masc* (Figure 1A), and 13 in *P. xylostella Masc* [34]. *A. ipsilon Masc* yields one constitutively spliced transcript only [33], whereas *Masc* from *B. mori* [44], *H. armigera* (Figure 1), and *P. xylostella* [34] produce not only one constitutively spliced transcript but also one alternatively spliced transcript. Moreover, the alternatively spliced transcript is generated by the presence of an alternative 3′ splicing site in exon 9 of *BmMasc*, the retention of intron 1 in *HaMasc* (Figure 1), and the skipping of exon 5–12 in *PxMasc* [34], respectively.

Despite the above divergences in gene structure, alternative splicing type, and amino acid sequence, the data from the current study on *HaMasc*’s chromosomal affiliation (Figure 4), expression profiles (Figure 5), and functions (Figure 6 and Figure 7) reveal that HaMasc is functionally homologous to BmMasc and other identified lepidopteran Masc proteins. Similar to *BmMassc* [6] and *PxMasc* [34], *HaMasc* is a Z chromosome-linked protein-coding gene, since its genomic copy number was two times higher in males than in females (Figure 4). The highest expression of *HaMasc* in the embryonic stage (12 h and 24 h after oviposition) (Figure 5) not only corresponds to the developmental expression profiles of *BmMasc* [6] and *PxMasc* [34] but also agrees with the notion that sex is determined developmentally upon embryogenesis [1,2,3]. Sexually, *HaMasc* expression, similar to *BmMasc* expression [6], was higher in males than in females (Figure 5), indicating that an unidentified primary signal, resembling Fem piRNA in female silkworms, diminishes *HaMasc* expression in female cotton bollworms. Functionally, *HaMasc* is required for the regulated male-specific splicing of *Hadsx* because RNAi suppression of *HaMasc* induced a *Hadsx* splicing shift from male-specific isoforms only to female-specific isoforms plus a reduced abundance of male-specific isoforms in the *H. armigera* male embryo cell line QB-Ha-E-1 (Figure 6) and male pupae (Figure 7B). Furthermore, the knockdown of *HaMasc* starting from the third instar larvae resulted in a series of male-specific phenotypic defects including smaller male pupa and testes, fewer but larger/longer spermatocytes and sperm bundles, delayed pupation and internal fusion of the testes and follicles (Figure 7). The delay of testes/follicle fusion and spermatogenesis, the major remaining steps of male gonad development post-*HaMasc* depletion from the third instar larvae plus the observed *HaMacs*-regulated shift of *Hadsx* splicing in vitro (Figure 6) and in vivo (Figure 7B) demonstrate that *HaMasc* functions as a male-determinant targeted by the unidentified female-specific primary signal in the *H. armigera* sex-determination cascade.

While several lepidopteran Masc proteins have been functionally studied [32,33,34], this study reports for the first time that *Masc* knockdown suppresses male growth and body size/weight (Figure 7D,E,G). This is not a surprise since at least two upstream sex-determination pathways genes have been found to promote growth, development, and body size of the sex in which they are expressed [45,46,47]. In male *B. mori*, the loss of *BmImp*, the autoregulatory key gene immediately downstream of *BmMasc* [25], suppresses larval growth and reduces body size [46]. Additionally, in male *B. mori*, lack of the CCCH-type zinc finger gene *Bmznf-2*, a recently discovered redundant masculinizer of the CCCH-type zinc finger gene *BmMasc* [48], results in developmental delay and smaller body sizes of male larvae [47]. Given the functional similarity of *Masc* with *Bmznf-2*, its regulatory relationship with *BmImp,* and involvement in dosage compensation [41,42,43], *HaMasc* may regulate male growth and body size/weight by itself, indirectly via its immediate downstream gene, i.e., the *H. armigera* homolog of *BmImp*, or by dosage compensation. Additional experiments are needed to resolve the three possibilities.

The female-specific primary signal in *H. armigera* is most likely a non-coding RNA belonging to piRNA, micro-RNA, or long non-coding RNAs. This is because the female-specific W chromosome in Lepidoptera is packed with transposons that can be transcribed into non-coding RNAs [6,49,50,51]. Our recent characterization of the first W-specific protein-coding gene *GUW1* from *H. armigera* [35] suggests that the W-specific *GUW1* may function as the primary signal to minimize *HaMasc* expression in female cotton bollworms. Further experiments are required to examine these two possibilities.

## 4. Materials and Methods

### 4.1. Insects

The *H. armigera* strain used in this study was collected from tobacco fields in Xuchang City (Henan Province, China) and was reared under laboratory conditions (28 °C with a photoperiod of 14L:10D, and 75 ± 5% R.H.) [52]. Larvae were reared individually in disposable plastic cups on a casein-based artificial diet as previously described [53]. Cotton wicks soaked with a 10% honey solution were provided for adult moths to supplement adult feeding.

### 4.2. DNA and RNA Isolation and Reverse Transcription

Three male and three female genomic DNA samples of *H. armigera* were extracted from 100 mg tissues of three frozen male and female pupae, respectively, using the same procedure as described previously by Li et al. [54]. The purified DNA samples were dissolved in double-distilled water (ddH_2_O), measured using the NanoDrop 1000 (Thermo Scientific, Logan, UT, USA), and stored at −20 °C for subsequent genomic PCR-gel analysis and genomic quantitative PCR (qPCR) analysis of *HaMasc*.

RNA samples were extracted from a variety of insect tissues throughout its life cycle, including eggs, larvae of the first to the last instars (separation of male and female larvae after third instars), male pupa, female pupa, and head, thorax, abdomen, leg, and wings of newly emerged male and female adults of *H. armigera*. Total RNA was extracted with a Trizol reagent (Invitrogen, Carlsbad, CA, USA) following the manufacturer’s instructions. The RNA concentration was measured by the NanoDrop 1000 (Thermo Scientific). Before reverse transcription, the RNA was treated with DNase I (Promega, Madison, WI, USA) with an RNase inhibitor (Thermo Scientific) following the manufacturer’s instructions to avoid genomic DNA (gDNA) contamination. Two µg RNA were reverse transcribed into the first strand of cDNA using the Quant Reverse transcriptase kit (Tiangen Biotech, Beijing, China). The primer used was a mixture of the 6-mer random primer and the oligo(dT) primer. The cDNA products were diluted using ddH_2_O and stored at −80 °C for subsequent RT-PCR cloning and/or RT-qPCR analysis amplification of *H. armigera Masc* (*HaMasc*).

### 4.3. Cloning of H. armigera Masc cDNA Sequence

A contig containing the partial cDNA sequence (Transcriptome annotated number: asmbl_65189) of *HaMasc* was retrieved by TBLASTN search of an *H. armigera* larval transcriptome dataset [30] using BmMasc amino acid sequences (NP_001296506.1) as a query sequence. This partial sequence was further verified by RT-PCR cloning using the aforementioned egg cDNA as the template and the primers Harm-mascF2 and Harm-mascR2 (Table S1; Figure 1) designed based on the retrieved contig asmbl_65189. The PCR conditions were 95 °C for 5 min; 40 cycles of 10 s at 98 °C, 15 s at 60 °C, and 3 min at 68 °C; and the final extension at 68 °C for 5 min. The resultant PCR product was cloned into a pGEM-T Easy vector (Promega, Madison, WI, USA) and sequenced by Sangon Biotech (Shanghai, China). To obtain the full-length cDNA sequence of *HaMasc*, we performed 3′ and 5′ RACE reactions using egg RNA as the template and the SMARTer RACE cDNA Amplification Kit (Clontech, Mountain View, CA, USA) following the manufacturer’s instructions. The general and *HaMasc*-specific primers used in the 3′ and 5′ RACE are listed in Table S1. The two full-length isoforms of *HaMasc* transcripts have been deposited in the GenBank database (accession number: MH844486 and MH844487).

### 4.4. Analysis of HaMasc Sequence

The open reading frames (ORFs) of the two full-length cDNA sequences of *HaMasc* were predicted using the ORF search tool within the Clone Manager 8 Software (Scientific and Educational Software, Durham, USA). The similarity of multiple Masc protein sequences was analyzed using DNAMAN (Version 8.0, Lyn-non Biosoft, Canada). The exons and introns of *HaMasc* were identified by aligning its cDNA sequences to its *HaMasc*’s genome sequence (Li et al., unpublished genome data) and the gene structure was generated using the Gene Structure Display Server 2.0 [55]. The conserved domains among known Masc proteins were identified using the multiple sequence alignment software Mega X [56], NCBI sequence analysis tools CDD (NCBI’s conserved domain database) [57], and the GeneDoc multiple sequence alignment tool [58].

Masc amino acid sequences of *Agrotis ipsilon* (Sequence reference [33], *Artemia franciscana* (accession number: ARB66312.1), *Artemia parthenogenetica* (accession number: ARB66313.1), *B. mori* (accession number: NP_001296506.1), *Trilocha varians* (accession number: BAS02075.1), and *Ostrinia fumacalis* (accession number: BAS02074.1) were obtained from the NCBI protein database. The Masc protein sequences were aligned using the ClustalW tool in MEGA [56] and the phylogenic tree was constructed using the neighbor-joining method.

### 4.5. Genomic PCR and qPCR Analysis of HaMasc

The male and female pupal DNA samples obtained above were used as the templates for qPCR analysis of *HaMasc*, two autosomal genes *Elongation factor 1 alpha* (*EF-1α*; GeneBank accession number: FJ768770.1), *beta-Actin* (*β-actin*; GeneBank accession number: EU527017.1), and one W chromosome gene *GUW1* [35]. The qPCR reactions for each of the four genes contained 1 μL of male or female DNA (100 ng), 10 μL 2 × SuperReal PreMix Plus (SYBR Green, Tiangen), 0.4 μL of ROX Reference Dye, 1 μL gene-specific forward primer, and 1 μL gene-specific reverse primer (see Table S1 and Figure 1), and 7.6 μL RNase-free water. The qPCR cycling conditions were the same as previously described for RT-PCR cloning of the *HaMasc* partial sequence.

The two DNA samples were also used as the templates for PCR-gel analysis of *HaMasc* with the primer pair Hamasc-1220F and Hamasc-1352R (Table S1) and *EF-1α* with the primer pair EF-F and EF-R (Table S1). The genomic PCR reactions for each of the two genes included 1 μL (100 ng) of male or female DNA, 1 μL PrimeSTAR GXL DNA polymerase, 10 μL 5 × PCR buffer, 2.5 μL gene-specific forward primer, 2.5 μL gene-specific reverse primer, and 33μL RNase-free water. The PCR cycling conditions were the same as what was used for the RT-PCR cloning of the *HaMasc* partial sequence. The resultant PCR products were fractioned on 1.2% agarose gel and visualized by ethidium bromide fluorescent staining.

### 4.6. RT-qPCR Analyses of HaMasc Expression

The cDNA samples prepared above were used as the templates for the RT-qPCR analysis of *HaMasc* expression in eggs, larvae of the first to the last instars, male pupa, female pupa, and different body parts of newly emerged male and female adults of *H. armigera.* The RT-qPCR reactions contained 1 μL of each cDNA sample, 10 μL 2 × SuperReal PreMix Plus (SYBR Green, Tiangen), 0.4 μL of ROX Reference Dye, 1 μL gene-specific forward primer, 1 μL gene-specific reverse primer, and 7.6 μL RNase-free water. The RT-qPCR running conditions were 95 °C for 5 min; 40 cycles of 10 s at 98 °C, 15 s at 60 °C, and 3 min at 68 °C; and the final extension at 68 °C for 5 min on an ABI 7500 real-time PCR instrument (Applied Biosystems, Foster City, CA). The gene-specific forward and reverse primers for RT-qPCR analyses of *HaMasc* and the two reference genes *EF-1α* and *β-actin* are listed in Table S1. Each developmental stage or body part had three independent biological replicates; RT-qPCR analysis of each biological replicate was repeated three times. The normalized expressions of *HaMasc* in each developmental stage or body part were calculated using the 2^−ΔΔCt^ method [59].

### 4.7. RNAi Knockdown of HaMasc in H. armigera Embryo Cell Line and Larvae

#### 4.7.1. RNAi Knockdown of *HaMasc* in *H. armigera* Cell Line

Three 3’end 2´-O-Methyl-modified and 5´end 5´-Cholesterol small interfering RNAs (siRNA) targeting at different regions of *HaMasc* (see Figure 1) and one negative control siRNA (NC siRNA) were designed and synthesized by Ribobio Biotech (Guangzhou, China) to knock down the endogenous *HaMasc* in *H. armigera* larvae and embryo cell line QB-Ha-E-1 [60], respectively. QB-Ha-E-1 cells were routinely cultured at 28 °C with Grace’s Insect Medium (Gibco/Life Technologies, New York, NY, USA) supplemented with a 10% fetal bovine serum (Gibco/Life Technologies, New York, NY, USA), 50 U/mL penicillin, and 50 μg/mL streptomycin (HyClone, Thermo Scientific, Logan, UT, USA). QB-Ha-E-1 cells seeded onto 12-well plates grew to 70% confluence, we transfected each well of cells with 1 μL of a 1:1:1 mixture of the three *HaMasc* siRNAs (total 3 siRNAs 20 pmol) or equal amount and volume of NC siRNA using the transfection agent Lipofectamine 3000 (Thermo Scientific, Logan, UT, USA) according to the product manual. Briefly, we added 2 μL of Lipofectamine 3000 and 1 μL of *HaMasc* siRNA mixture or NC siRNA into a 1.5 mL microcentrifuge containing 50 μL of serum-free Grace’s medium, mixed it well, incubated it for 15 min at room temperature, and transferred the whole mixture to one well of cells. The medium in each well was gently replaced with fresh completed Grace’s medium 12 h post-transfection. After another 72 h, the cells in each well were harvested, flash-frozen in liquid nitrogen, and stored at −80 °C for subsequent RNA extraction, and RT-PCR and/or RT-qPCR analyses of *HaMasc* and *H. armigera dsx* (*Hadsx*). RT-PCR and RT-qPCR analyses of *HaMasc* were performed as described above, while RT-PCR gel analysis of *Hadsx* transcript isoforms was conducted under the conditions described later (see below).

#### 4.7.2. RNAi Knockdown of *HaMasc* in *H. armigera* Larvae

Third instar larvae within 6 h of molting were individually microinjected with a microinjector consisting of a 5 μL syringe connected to a capillary glass needle (Figure S2) from their dorsal intersegmental membrane between abdominal segment 2 and 3 with 0.1 μL ddH_2_O, NC siRNA (20 pmol/μL), or a 1:1:1 mixture of the three *HaMasc* siRNAs (total 3 siRNAs concentration 20 pmol/μL) [61]. Three replicates of 30 larvae each (N = 90) were injected for each control or siRNA treatment. The injected larvae were then reared in 1 OZ plastic cups (1 larva/cup) containing the corresponding KOH-free diets supplemented with 20 pmol/g diets of ddH_2_O, NC siRNA, or a 1:1:1 mixture of the 3 *HaMasc* siRNAs (see their target regions in Figure 1B) [62]. Three male and three female fourth instar larvae per replicate were randomly flash-frozen with liquid nitrogen and stored at −80 °C for subsequent RNA extraction, RT-PCR, and RT-qPCR analyses of *HaMasc* 72 h post-microinjection.

The remaining 24 larvae of each replicate were then transferred to cups containing the corresponding freshly made ddH_2_O diets, NC siRNA diets, or *HaMasc* siRNAs diets. The old diets in each cup were replaced with the corresponding fresh ones every 72 h [61,62]. Larvae were reared on the respective diets until pupation or up to two weeks. Pupae were individually sexed, photographed, weighed, flashed-frozen in liquid nitrogen, and stored at −80 °C for the subsequent analysis of *HaMasc* expression and *Hadsx* splicing as well as the dissection and hematoxylin-eosin staining of pupal testes after development at 28 °C for 3 d. The survival and pupation rates of male and female individuals two weeks post-injection were recorded.

### 4.8. Hematoxylin-Eosin (HE) Staining of Pupal Testes

Pupal samples from each control or siRNA treatment were obtained from the −80 °C freezer, thawed at room temperature, and individually dissected to get testes. Testes were individually weighed, fixed overnight in Bouin’s fluid [63], photographed, paraffin-embedded, sectioned into 10 μm-thick slices with a Leica RM2235, and stained using a mixture of hematoxylin and eosin solution (Sangon Biotech, E607318). The stained testis sections were observed and photographed under a microscope (CX53, Olympus, Japan).

### 4.9. RT-PCR Gel Analysis of Female and Male-Specific Isoform of Hadsx Transcript

After *HaMasc* was knocked down in 3-d-old male pupae or the male cell line QB-Ha-E-1, sex-specific splicing isoforms of *H. armigera doublesex* (*Hadsx*) transcripts were detected by RT-PCR. The RNA extraction, reverse transcription, and PCR reaction procedures and experimental conditions were the same as described above for *HaMasc*. The primers used for *Hadsx* RT-PCR are listed in Table S1 (Ha-DX-exo2F and Ha-DX-exo6R, which produced a 419 bp amplicon in control males but four amplicons of 668, 683, 797, and 812 bp in control females. *EF-1α* (primer list in Table S1) was used as the endogenous control.

## 5. Data Analysis

One-way analysis of variance (ANOVA) followed by Tukey’s HSD tests was performed to determine differences in *HaMasc* expression among different tissues, developmental stages, and larvae with different siRNA injection/feeding treatments, as well as in pupation rate and pupa and testis weights among larvae with different siRNA injection/feeding treatments. We conducted independent *t*-tests to compare the expressional differences of *HaMasc* between cells transfected with an NC siRNA or a *HaMasc* siRNA mixture. All statistical analyses were performed in SPSS version 19.0 (SPSS Inc., Chicago, IL, USA).

## Figures and Tables

**Table 1:** Amino acid sequence identity among lepidopteran Masc proteins.

	BmMASC	TvMASC	OfMASC	AiMASC	HaMASC	PxMASC
BmMASC	100.00					
TvMASC	54.99	100.00				
OfMASC	22.48	22.29	100.00			
AiMASC	21.38	19.37	30.71	100.00		
HaMASC	25.00	22.52	34.01	62.57	100.00	
PxMASC	19.74	17.01	22.30	19.76	21.14	100

## Data Availability

All data supporting results are included in the Supplementary Files.

## Supplementary Materials

The following are available online at https://www.mdpi.com/article/10.3390/ijms22168650/s1.
