# Supplementary material for: Identification and Characterization of the Masculinizing Function of the Helicoverpa armigera Masc Gene
Source: Int J Mol Sci. 2021 Aug 11;22(16):8650. doi: 10.3390/ijms22168650 (PMC8395511; doi:10.3390/ijms22168650)
Supplement: Supplementary file 1 [file ijms-22-08650-s001.zip › ijms-1297653-supplementary.pdf]

Table S1. Oligos used in this study.

Primer name	Sequence (5' to 3')	Length (bp)	Efficiency (%)	Tm (°C)
Primer for 5'RACE and 3'RACE of *HaMasc*				
Harm-mascF2	CCTGTTTGGCTTGATTTGAATACCAAG	1966		60
Harm-mascR2	CTCGTCAGATTTATTGGAATGGGGGA			
Hamasc5'GSP1	CGTGGGTCTGTTGGAGCTACTGATGG			60
Hamasc5'GSP2	CAAAGGCGGCTGACGGCCATTTTGC			58
Hamasc3'GSP1	TCAGCCGCATCACTATCCCCCATTCC			60
Hamasc3'GSP2	GTGGGTGTAACTCAAGCACCTTCGCG			60
Primer for *HaMasc* DNA and cDNA RT-PCR and RT-qPCR				
β-ACT-F	CCTGGTATTGCTGACCGTATGC	144	97.87	60
β-ACT-R	CTGTTGGAAGGTGGAGAGGGAA			
EF-F	GAAGTCAAGTCCGTGGAGATG	171	95.15	60
EF-R	GACCTGTGCTGTGAAGTCG			
qRT-mascF2	AGGTCCAATCCTGTCACTCATTC	254(DNA)	102.01	60
qRT-mascR2	CTGTTGTTTACGTTACTAGTGCTCG	169(cDNA)		
Semi-mascF2	ACCGTGCCGAAACAGTTCCTACT	397		58
Semi-mascR2	GTGCTCGGGTATCTGCTGGCACT			
Ha-DX-exo2F	GGTGCTCGTGATCCTCAACTATG	419/668/683 /797/812		60
Ha-DX-exo6R	CTTAACAGATCTAGCACTAGGTGTCCTG			
Hamasc-1220F	AGTTGGAGTGCGAGAGGGAATATCA			60
Hamasc-1352R	GGAACTGTTTCGGCACGGTAGATGTA			
qRT-masc1F	ACATAGTTTGCTGCGGTAAGTTTAC	165	91.22	60
qRT-masc1/2R	CTACTGATGGTGCCGACTGTTGC			
qRT-masc2F	ACATAGTTTGCTGCGTTCATGAGTAA	85	90.64	60
qRT-masc1/2R	CTACTGATGGTGCCGACTGTTGC			

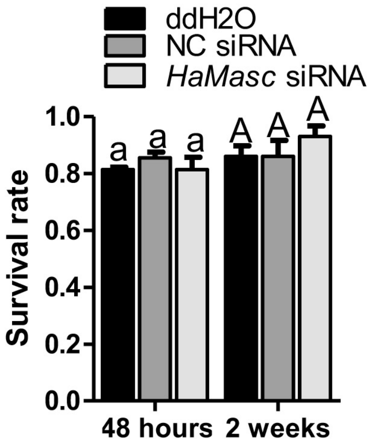

Figure S1. Impacts of *HaMasc* siRNA mixture on *H. armigera* larvae survival. ddH2O, NC siRNA, *HaMasc* siRNA was different treat group. Bars with different letters are significantly different ($P < 0.05$, One-way ANOVA followed by Tukey's HSD test).

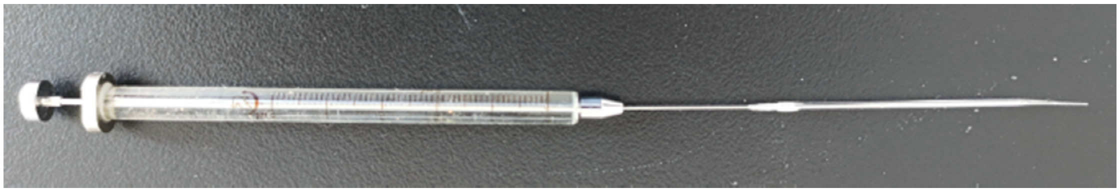

Figure S2 siRNA injection equipment is composed of (A) a capillary glass needle and (B) a 5 μL syringe.
